# Adenylyl Cyclase Signaling in the Developing Chick Heart: The Deranging Effect of Antiarrhythmic Drugs

**DOI:** 10.1155/2014/463123

**Published:** 2014-06-23

**Authors:** Lucie Hejnova, Klara Hahnova, Radka Kockova, Jarmila Svatunkova, David Sedmera, Jiri Novotny

**Affiliations:** ^1^Department of Physiology, Faculty of Science, Charles University in Prague, 128 43 Prague, Czech Republic; ^2^Institute of Physiology, Academy of Sciences of the Czech Republic, 142 20 Prague, Czech Republic; ^3^Institute of Anatomy, First Faculty of Medicine, Charles University in Prague, 128 00 Prague, Czech Republic

## Abstract

The adenylyl cyclase (AC) signaling system plays a crucial role in the regulation of cardiac contractility. Here we analyzed the key components of myocardial AC signaling in the developing chick embryo and assessed the impact of selected *β*-blocking agents on this system. Application of metoprolol and carvedilol, two commonly used *β*-blockers, at embryonic day (ED) 8 significantly downregulated (by about 40%) expression levels of AC5, the dominant cardiac AC isoform, and the amount of Gs*α* protein at ED9. Activity of AC stimulated by forskolin was also significantly reduced under these conditions. Interestingly, when administered at ED4, these drugs did not produce such profound changes in the myocardial AC signaling system, except for markedly increased expression of Gi*α* protein. These data indicate that *β*-blocking agents can strongly derange AC signaling during the first half of embryonic heart development.

## 1. Introduction

Cardiovascular diseases represent frequent life-threatening health problems in the western society and they can also be encountered during pregnancy [1, 2]. It is evident that the need of expanded use of cardiac medication goes hand in hand with the increasing age of pregnant women. Nowadays, *β*-blockers are the most commonly used agents for treatment of arrhythmias, ischemic heart disease, heart failure, and hypertension [3]. Nevertheless, there is only sparse information concerning potential harmful effects of the *β*-blockers on fetal development and child health when used in pregnancy. Some recent findings indicate that children of mothers treated with *β*-blockers during pregnancy may be at higher risk for cardiovascular defects [4–6]. However, the embryotoxic mechanisms of these compounds are still not well understood.

The chick embryonic heart has proven to be a good model for studying developmental physiology of the cardiovascular system and it can also be useful for exploring cardiotoxicity of catecholamines and *β*-blocking agents [7]. In the chick embryo, the onset of sympathetic cardiac innervation occurs at about embryonic day (ED) 9 and this system is functional by ED16 [8]. During subsequent stages of development, the number of myocardial *β*-adrenergic receptors increases, reaching adult values on postnatal day 7 [9]. However, autonomic receptor-mediated effector mechanisms seem to be present in the heart before there is functional innervation. The ability of exogenous noradrenaline to elicit acceleration of heart rate has been observed already in the early stages of chick embryo development [10]. Interestingly, the preneural period of heart development is characterized by the presence of relatively high levels of catecholamines, which may be secreted by the developing adrenal medulla or sympathetic chain ganglia [11, 12].

Some early experiments with catecholamines and *β*-blockers revealed that the chick heart is more resistant to these drugs in early embryonic stages than in later stages of prenatal development [13]. Our previous study focused on determining the effect of metoprolol and carvedilol, two *β*-blockers frequently used in clinical practice, indicated that administration of these drugs at ED8 may cause much more pronounced decrease in heart rate when compared to treatment at ED4 [14]. Interestingly, this effect was mediated by lower number of *β*-adrenergic receptors (*β*-ARs) in older chick embryos. In contrast to metoprolol, carvedilol lowered the number of myocardial *β*-ARs, but metoprolol reduced heart rate more effectively than carvedilol [14].

It is well known that *β*-ARs receptors transfer information through their cognate G proteins to adenylyl cyclase (AC), and this signaling system is crucially involved in the regulation of cardiac function [15, 16]. The main aim of our present work was to characterize the major components of the chick myocardial AC signaling system during embryonic development and to assess the potential impact of *β*-blockers on its function.

## 2. Materials and Methods

### 2.1. Materials

[*α*-^32^P]ATP was purchased from Izotop (Budapest, Hungary), [^3^H]cAMP from ARC (St. Louis, MO, USA), and scintillation cocktail EcoLite from MP Biomedicals (Santa Ana, CA, USA). Acrylamide and bisacrylamide were from SERVA (Heidelberg, Germany), and aluminum oxide 90 (neutral, activity I) was from Merck (Darmstadt, Germany). All other chemicals were from Sigma (St. Louis, MO, USA) and they were of the highest purity available. AC5 antibody was from Abcam (Cambridge, UK) and Gs*α* and actin antibodies were from Santa Cruz Biotechnology, Inc. (Dallas, TX, USA). Preparation and characterization of Gi*α*(1,2) antibody were described previously [17].

### 2.2. Chick Embryo Incubation and Drug Application

Fertilized white Leghorn chicken eggs were incubated with their blunt end up in forced draft incubator at 38°C and 75% humidity up to embryonic days (ED) 4–18. The eggs were turned automatically every 4 h. At ED4 or ED8 or ED4 + ED8, 200 *μ*L metoprolol (1 mg/mL) or 50 *μ*L carvedilol (1 mg/mL) dissolved in normal saline was administered intra-amniotically through a small opening made in the egg shell. More specifically, the eggs were divided into seven groups. Whereas the first group received metoprolol and the second group carvedilol at ED4, the third and fourth group received, respectively, metoprolol and carvedilol only at ED8. The fifth group received metoprolol at both ED4 and ED8 (ED4 + ED8), and the sixth group received carvedilol at both ED4 and ED8 (ED4 + ED8). Normal saline was administered to the seventh group (control, ED9) at both ED4 and ED8. After finishing injecting, all the eggs were sealed and further incubated until ED9.

### 2.3. Preparation of Crude Membranes

Chick hearts (normal at various developmental stages and ED9 with and without treatment with beta-blockers at ED4, ED8, and ED4 + ED8) were homogenized in TMES buffer containing 20 mM Tris-HCl, 3 mM MgCl_2_, 1 mM EDTA, and 250 mM sucrose (pH 7.4) supplemented with protease inhibitor cocktail (Roche Diagnostics), using Potter-Elvehjem glass-teflon homogenizer on ice. Coarse cell debris and nuclei were removed by low-speed centrifugation (600 g, 10 min, 4°C), and membranes were then pelleted by centrifugation at 50,000 g for 30 min at 4°C. The pellets were resuspended in TME buffer (20 mM Tris-HCl, 3 mM MgCl_2_, and 1 mM EDTA; pH 7.4), aliquoted into Eppendorf tubes, snap-frozen in liquid nitrogen, and stored at −80°C.

### 2.4. Assessment of Adenylyl Cyclase Activity

Adenylyl cyclase (AC) activity in myocardial membranes was determined by measuring the conversion of [*α*-^32^P]ATP to [^32^P]cAMP according to the method of Salomon et al. [18]. Myocardial membranes (20 *μ*g of proteins) were incubated in the reaction mixture (100 *μ*L) containing 48 mM Tris-HCl buffer (pH 7.4), 100 mM NaCl, 2 mM MgCl_2_, 20 *μ*M GTP, 0.8 mg/mL BSA, 5 mM phosphoenolpyruvate, 3.2 U of pyruvate kinase, 40 *μ*M 3-isobutyl-1-methylxanthine, 0.1 mM cAMP, and about 10 000 cpm [^3^H]cAMP as a tracer. Stimulated AC activity was measured after addition of 10 *μ*M isoprenaline or 10 *μ*M forskolin. After 1 minute preincubation 0.4 mM ATP was added along with 200,000 cpm [*α*-^32^P]ATP and incubation proceeded for 20 min at 30°C. The reaction was stopped by addition of 200 *μ*L 0.5 M HCl and heating at 100°C for 5 min. Samples were neutralized by 200 *μ*L 1.5 M imidazole. Separation of newly formed [^32^P]cAMP was performed by using dry alumina column chromatography as described by White [19]. Column recovery was usually about 70–75%.

### 2.5. Electrophoresis and Western Blotting

Samples of myocardial membranes were solubilized in Laemmli buffer and loaded (30 *μ*g per lane) on standard 10% acrylamide gels for SDS-PAGE. After electrophoresis, the resolved proteins were transferred to nitrocellulose membrane (Schleicher & Schuell), blocked with 5% nonfat dry milk in TBS buffer (10 mM Tris and 150 mM NaCl; pH 8.0) for 1 h, and then incubated with relevant primary antibodies overnight at 4°C. After three 10 min washes in TBS containing 0.3% Tween 20, the secondary anti-rabbit IgG labeled with horseradish peroxidase was applied for 1 h at room temperature. After another three 10 min washes in TBS-Tween, the blots were visualized by enhanced chemiluminescence technique according to the manufacturer's instructions (Pierce Biotechnology, Rockford, IL, USA). To ensure equal protein loading on the gel, the membranes were stripped and reprobed with actin antibody. The immunoblots were scanned and quantitatively analyzed by ImageQuant TL software (Amersham Biosciences). Relative expression levels of AC5, Gs*α*, and Gi*α* were always normalized to the corresponding expression of actin.

## 3. Results

### 3.1. Adenylyl Cyclase Activity

**Figure 1:**
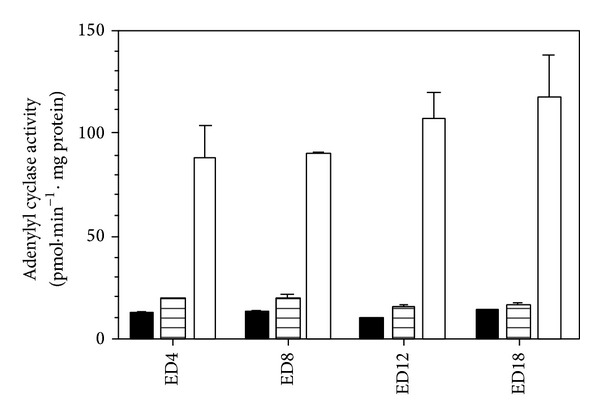
Adenylyl cyclase activity in the chick embryonic heart during ontogenesis. Basal (solid bars), isoprenaline-stimulated (hatched bars), and forskolin-stimulated (open bars) AC activity was monitored in myocardial membrane preparations from different stages of embryonic development. All values represent the mean ± SEM of three determinations measured in triplicate.

Activity of AC in myocardial preparations from chick embryos did not significantly differ between ED4 and ED18 (Figure 1). Whereas the *β*-adrenergic agonist isoprenaline increased the basal AC activity by about 40–45%, forskolin, a direct activator of the AC catalytic subunit and Gs protein, augmented the enzyme activity up to 10-fold. However, the extent of the stimulatory effects of both of these activators did not significantly differ between samples prepared from different developmental stages of the chick embryo. It is worth noting that there was a slight downward tendency in isoprenaline-stimulated AC activity in the later stages of embryonic development.

**Figure 2:**
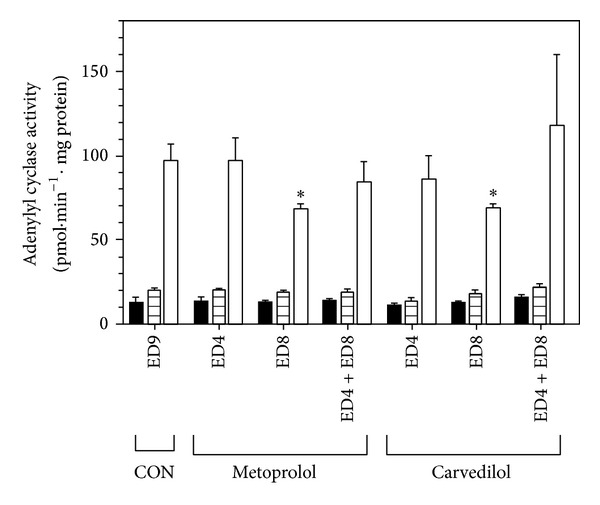
Effect of *β*-blockers on adenylyl cyclase activity in the chick embryonic heart. A dose of metoprolol or carvedilol was injected into chick embryos either at ED4 (ED4 Met or ED4 Car) or at ED8 (ED8 Met or ED8 Car) or at both ED4 and ED8 (ED4 + ED8 Met or ED4 + ED8 Car). Control embryos (CON) received normal saline at ED4 and ED8. All the embryos were incubated until ED9. Subsequently, all ED9 embryos (both control and those affected by *β*-blockers) were used for preparation of myocardial membranes and determination of basal (solid bars), isoprenaline-stimulated (hatched bars), and forskolin-stimulated (open bars) AC activity. Data shown are means ± SEM of triplicate measurements. ∗ Indicates a statistically significant difference between the indicated group affected by *β*-blockers and control (*P* < 0.05).

In the next series of experiments we investigated the impact of *β*-blockers applied during early developmental stages on myocardial AC activity assessed at ED9. Neither metoprolol nor carvedilol injected into the developing chick embryos at ED4 or ED4 + ED8 altered AC activity at ED9 but both of these drugs significantly suppressed forskolin-stimulated AC activity when administered at ED8 (Figure 2).

### 3.2. Developmental Expression of Adenylyl Cyclase and G Proteins

**Figure 3:**
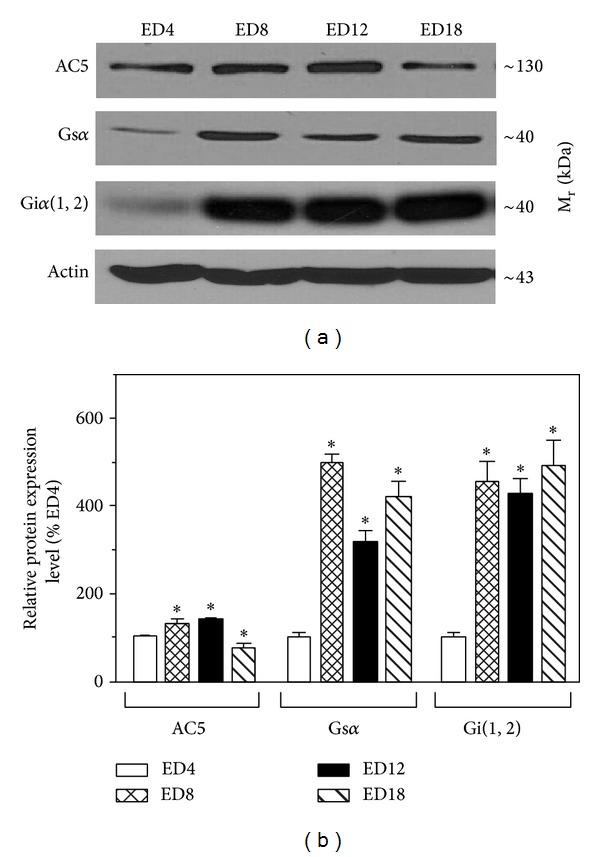
Expression of adenylyl cyclase and G protein subunits in the developing chick heart. Proteins from myocardial membrane preparations derived from different developmental stages of the chick embryo were resolved by SDS-PAGE, transferred to nitrocellulose membranes, and probed with specific antibodies to AC5 isoform and *α* subunits of Gs and Gi(1,2) proteins. After stripping, the membranes were reprobed with actin antibody to control for differences in protein loading. Representative blots are shown, each of three independently performed experiments (a) and the graphical representation of relative expression levels (means ± SEM) of AC5, Gs*α*, and Gi*α* normalized to the corresponding expression of actin (b). ∗ Indicates significantly different expression of the individual proteins in samples from ED8, ED12, and ED12 as compared to ED4 (*P* < 0.05).

**Figure 4:**
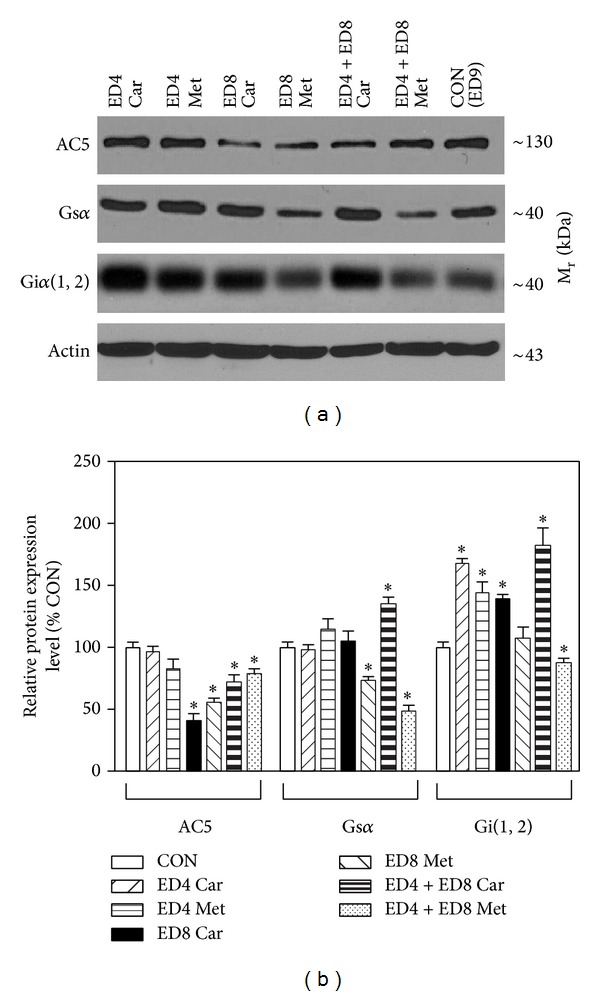
Effect of *β*-blockers on expression of adenylyl cyclase and G protein subunits in the chick embryonic heart. Samples of myocardial membrane proteins prepared from ED9 chick embryos, both control (CON) and previously treated with metoprolol (Met) or carvedilol (Car) at ED4, ED8, or ED4 + ED8, were resolved by SDS-PAGE, transferred to nitrocellulose membranes, and probed with specific antibodies to AC5 isoform and *α* subunits of Gs and Gi(1,2) proteins. After stripping, the membranes were reprobed with actin antibody to control for differences in protein loading. Representative blots are shown, each of three independently performed experiments (a) and the graphical representation of relative expression levels (means ± SEM) of AC5, Gs*α*, and Gi*α* normalized to the corresponding expression of actin (b). ∗ Indicates a statistically significant difference between the indicated group affected by *β*-blockers and control (*P* < 0.05).

The distribution of AC5 and *α* subunits of the stimulatory (Gs) and inhibitory (Gi) G proteins as the key components of the AC signaling system was determined in myocardial preparations from different developmental stages of the chick embryo by western blotting (Figure 3). The expression level of the dominant cardiac AC isoform, AC5, slightly increased during early stages of embryonic development, but it fell by over 30% between ED12 and ED18. Whereas AC5 expression was not affected by metoprolol or carvedilol at ED4, administration of these drugs at ED8 resulted in a marked decrease (by about 50%) in cardiac AC5 (Figure 4). When applied at ED4 + ED8, the *β*-blockers also reduced AC5 levels, but to a much lesser extent (by about 20%).

Immunochemical assessment of the developmental profile of selected G protein subunits indicated relatively very low expression levels of the *α* subunits of Gs and Gi proteins in the earliest stages of heart development and these levels increased dramatically between ED4 and ED8 (Figure 3). Thereafter, the expression of G protein *α* subunits remained relatively unchanged until ED18. When applied on ED4, metoprolol and carvedilol did not significantly affect Gs*α* levels in the developing chick heart (Figure 4). However, injection of metoprolol at ED8 and ED4 + ED8 led to a decline (by about 40–60%) in Gs*α* expression. Interestingly, no such effects were observed after treatment with carvedilol. By contrast, carvedilol applied at ED4 + ED8 increased Gs*α* expression by about 30%. Application of both *β*-blockers at ED4 and application of carvedilol at ED8 or ED4 + ED8 markedly increased (by about 60%) the amount of Gi*α* at ED9 (Figure 4). Treatment of chick embryos with metoprolol at ED8 or ED4 + ED8 did not elicit such profound changes in the content of myocardial Gi*α* protein.

## 4. Discussion

Heart function can be favorably modulated by *β*-blocking agents under some pathological conditions. However, there is also some evidence that these drugs may be potentially harmful to embryonic or fetal development when administered during pregnancy. In the present work we set to evaluate the impact of two widely used *β*-blockers, namely, metoprolol and carvedilol, on the key components and function of the myocardial adenylyl cyclase signaling system in the developing chick embryo.

Our current experimental results have indicated that, despite a perceptible decrease in the amount of the predominant isoform of cardiac adenylyl cyclase (AC5) between ED12 and ED18, the enzyme activity of AC is relatively stable in the course of chick embryo development, which is in line with some earlier findings [20, 21]. In parallel, rather incongruently, there was a marked increase in Gs and Gi protein levels between ED4 and ED8. However, a similar rise in myocardial Gi during the early development of the chick embryo was reported also by others [21]. Results of our previous study and some other studies indicated a clear drop in the number of myocardial *β*-adrenergic receptors during prenatal ontogenesis [14, 22, 23]. Together, these data suggest that *β*-ARs, G proteins, and AC are not coordinately regulated during development. It is also important to notice that partial loss of myocardial *β*-ARs in the later stages of embryonic development was reflected only by moderate insignificant diminution of isoprenaline-stimulated AC activity. This implies that there might have been sufficient receptor reserve (spare receptors) and that apparently not all the *β*-ARs must be necessarily coupled to AC to ensure the effective and reliable signal transmission. It has been reported previously that the amount of spare *β*-ARs may vary greatly between different tissues and species [24–26]. Furthermore, these findings can lead to the assumption that increased sensitivity to *β*-mimetics detected in the later stages of chick heart development is substantiated by a more efficient coupling of the *β*-ARs to their downstream effector AC.

We have previously observed that injection of *β*-blockers into the developing chick embryos at ED4, ED8, or ED4 + ED8 considerably reduced the number of myocardial *β*-ARs [14]. Results of our current research have shown that application of metoprolol and carvedilol at ED8, but not at ED4 or ED4 + ED8, can noticeably suppress AC activity stimulated by forskolin. However, basal and isoprenaline-stimulated AC activity was not significantly changed under these conditions. This seeming discrepancy can be at least partially explained by the decreased levels of G proteins, which were found in myocardial preparations from chick embryos affected with *β*-blockers at ED8. Forskolin can increase AC activity not only through direct interaction with the enzyme but also via Gs protein [27] and therefore lower amount of this G protein may well lie behind the diminished capacity of this agent to stimulate AC.

There is ample evidence to show that the regulatory mechanisms engaged in *β*-ARs-mediated signaling may substantially differ in the mature organism and during the prenatal period of development [28]. Whereas receptor signaling is controlled through the processes of desensitization and downregulation in adulthood, agonist-induced stimulation of *β*-ARs in the embryonic tissues fails to produce desensitization, and instead responsiveness increases. The peculiar regulatory mechanisms, which may include adaptive changes at the level of the receptors, G proteins, and AC, are instrumental for the necessary physiological adjustments during prenatal life and preparation for the postpartum period. It has become clear that there are unique developmental stages of susceptibility between species and embryonic exposure to harmful substances may lead to impairment of cardiac development and function [13, 29–32]. Our current research has revealed that application of the *β*-blockers metoprolol and carvedilol to the chick embryo during the first half of prenatal development can markedly derange myocardial *β*-ARs-mediated AC signaling. We have observed that *β*-blockers, which are used for their antiarrhythmic effects, may not always be beneficial but they can also affect the expression of myocardial *β*-ARs, G proteins, and AC as well as the function of this important signaling system during embryonic development. Therefore, physicians should be aware of potentially harmful side effects when prescribing these drugs during pregnancy.
